# Synthesis, spectroscopic studies, and antioxidant activities of novel thio/carbohydrazones and bis-isatin derivatives from terephthalaldehyde

**DOI:** 10.3906/kim-1910-13

**Published:** 2020-02-11

**Authors:** Halit MUĞLU, Hasan YAKAN, Temel Kan BAKIR

**Affiliations:** 1 Department of Chemistry, Faculty of Art and Science, Kastamonu University, Kastamonu Turkey; 2 Department of Chemistry Education, Faculty of Education, Ondokuz Mayıs University, Samsun Turkey

**Keywords:** Isatin, carbohydrazone, Schiff bases, DPPH, spectroscopic techniques

## Abstract

New bis(isatins-thio/carbohydrazones) based on Schiff bases were prepared from terephthalaldehyde biscarbohydrazone and 5-substituted isatins in the presence of a drop of sulfuric acid under reflux in ethanol. Terephthalaldehyde bis(thio/carbohydrazone) was synthesized by the reaction of (thio)/carbohydrazide and terephthalaldehyde in the presence of a few drops of acetic acid under reflux in ethanol. The structures of these synthesized compounds were determined using IR, ^1^H NMR, and ^13^C NMR spectroscopy and elemental analysis. The in vitro antioxidant activity of all the compounds was determined by the 1,1-diphenyl-2-picryl hydrazyl (DPPH.) free radical scavenging method. Compound 2 showed the best antioxidant activity.

## 1. Introduction

Schiff bases, including isatins, are known to have a broad range of pharmacological properties including anticonvulsant [1,2], antibacterial, antiviral [3–8], antioxidant [9–11], anti-HIV, and antifungal activity [3,4,6,7,12]. Substituted isatin-thio/carbohydrazones based on Schiff bases are commonly called β -isatin aldehyde-N,N′ - (thio)/carbohydrazones [13].

Isatin and its derivatives are a significant class of hetero compounds in organic chemistry. They have been reported to show several biological activities and have applications in pharmaceutical chemistry [6,14–16]. These have been reported as being antibacterial [15], antiviral [16], antifungal [6], antioxidant [17,18], anticonvulsant [19], antitubercular [20–22], and anti-HIV [21,23].

Reactive oxygen species (ROS) are formed in the body owing to radical oxygen during oxidative phosphorylation. Active ROS can damage DNA and cause mutation [24,25]. This damage is protected by many antioxidant molecules and antioxidant enzymes. Therefore, we report that new bis-isatins (thio)/carbohydrazones based on Schiff bases have been prepared from thio/carbohydrazones and 5-substituted isatins in the presence of a drop of H_2_SO_4_ under reflux in ethanol. All the synthesized compounds were elucidated using IR, ^1^H NMR, and ^13^C NMR spectroscopy and elemental analysis. The in vitro antioxidant activity of all the compounds was measured by the 1,1-diphenyl-2-picryl hydrazyl (DPPH.) free radical scavenging method and compared with ascorbic acid as the standard.

## 2. Experimental

### 2.1. Measurement and reagents

All reagents and solvents were purchased from Sigma, Aldrich, or Merck Chemical Company and were used without further purification. The solvents were of spectroscopic grade. Melting points were recorded using a Stuart Melting Point 30 apparatus and were left uncorrected. The elemental analysis was performed on a Eurovector EA3000-Single. A Bruker Alpha FT-IR spectrometer was used for infrared spectra. ^1^H and ^13^C NMR spectra were taken on a JEOL ECX-400 spectrophotometer (400 MHz) in DMSO-*d_6_* . Absorption measurements were recorded with a Shimadzu UV Pharmaspec 1700 spectrophotometer with a pair of identical quartz cuvettes of 1 cm path length.

### 2.2. Synthesis of terephthalaldehyde bis(thio/carbohydrazone) (1 and 2)

**Scheme 1:**
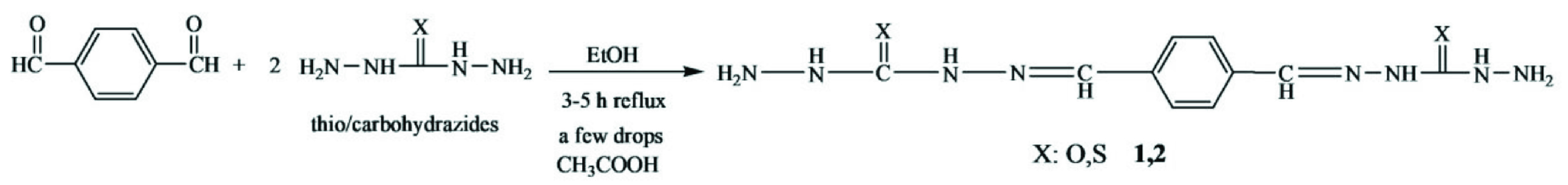
General synthesis of terephthalaldehyde bis(thio/carbohydrazone).

Thio/carbohydrazide (5.0 mmol) and terephthalaldehyde (2.5 mmol) with a few drops CH3 COOH as a catalyst, in an ethanol mixture (40 mL), were mixed and refluxed for 5 h. After the reaction was finished, the hot mixture was filtered. Then the filtrate was washed with ethanol. The formed solid was isolated and dried (Scheme 1).

### 2.3. 5-Substituted isatin-thio/carbohydrazones based on Schiff bases (3–10)

**Scheme 2:**
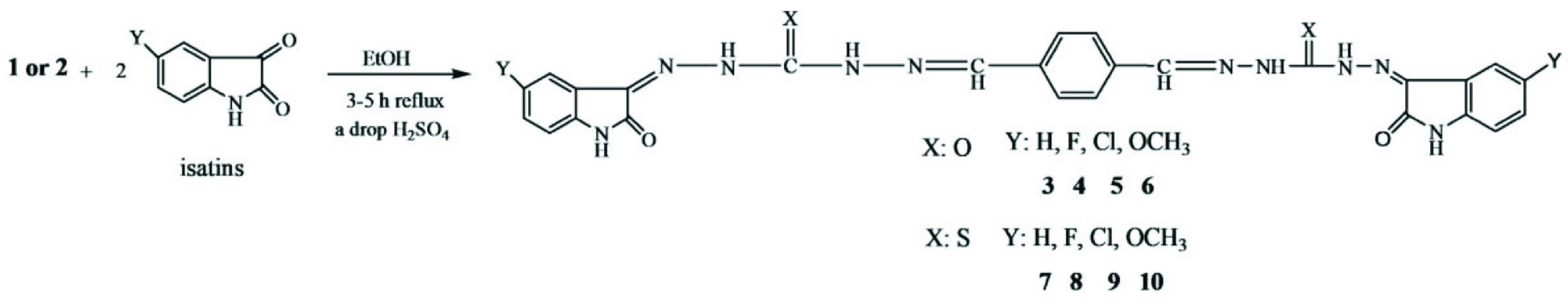
General synthesis of 5-substituted isatin-thio/carbohydrazones based on Schiff bases.

5-Substituted isatins (5.0 mmol) and compounds 1 and 2 (2.5 mmol) with a drop H_2_SO_4_ as catalyst in ethanol (40 mL) were mixed and refluxed for 5 h. After the reaction was finished, the solution was filtered and the filtrate was washed with ethanol. The formed solid was isolated and dried (Scheme 2).

### 2.4. Antioxidant activity

In order to evaluate the antioxidant activity of the newly synthesized compounds, we used DPPH in ethanol at a concentration of 55 μM. Stock solutions of the compounds (250 μM) were prepared in DMSO. To the previously prepared DPPH solution (4 mL) were added compound solutions of different concentrations (0.25, 0.50, 1.00, 2.50, and 5.00 μM) and enough ethanol to total 5 mL. This mixture was allowed to stand in a dark room at room temperature for 30 min and was then read at 517 nm against a blank [26–29].

The percentage inhibition of the free radical concentration for the sample compounds was calculated and compared to the standard ascorbic acid. Radical scavenging activity was expressed as a percentage of inhibition and calculated using the following formula:

Radical scavenging activity ( % ) = [(A_0_ – A_1_) / A_0_ ×100],

where A_0_ is the absorbance of the control (blank, without compound) and A_1_ is the absorbance of the compound [30].

In addition, IC_50_ values were calculated from the calibration curve. The IC_50_ value is defined as the concentration of the test compound required to obtain half maximum inhibition, and a lower IC_50_ value indicates more antioxidant activity [31].

## 3. Results and discussion

### 3.1. Physical properties

Ten of the synthesized compounds were new. The current experimental results for the physical properties, melting points, yields, and elemental analyses are summarized in Tables 1 and 2.

**Table 1:** The physical properties, melting points, and yields of the synthesized compounds.

Compounds	Molecular mass (g/mol)	Solubility	Melting point (°C)	Yields (%)
1	278.28	DMSO (+)	>350	98
2	310.40	DMSO (+)	>350	96
3	536.50	DMSO (+)	291–292	78
4	572.48	DMSO (+)	309–310	76
5	605.39	DMSO (+)	259–260	86
6	596.55	DMSO (+)	294–295	73
7	568.63	DMSO (+)	282–283	84
8	604.61	DMSO (+)	258–260	97
9	637.52	DMSO (+)	248–249	92
10	628.68	DMSO (+)	245–246	92

**Table 2:** The results of elemental analysis of the synthesized compounds.

Compounds	Calculated			Experimental		
	C%	H%	N%	C%	H%	N%
1	38.70	4.55	36.10	38.08	4.41	35.75
2	43.16	5.07	40.27	42.15	4.96	38.55
3	58.21	3.76	26.11	57.73	3.67	25.98
4	54.55	3.17	24.47	53.22	3.12	23.86
5	51.58	3.00	23.14	50.21	2.84	22.11
6	56.37	4.06	23.48	55.08	3.87	22.79
7	54.92	3.55	24.63	54.39	3.43	24.11
8	51.65	3.00	23.17	50.97	2.86	22.72
9	48.98	2.85	21.97	48.21	2.71	21.52
10	53.49	3.85	22.28	53.07	3.74	21.98

### 3.2. Vibrational frequencies

In the FT-IR spectrum of the synthesized compounds, the signal of the aldehyde group (-CHO, two bands) of the starting material was not observed near 2750–2650 cm^-1^ . Furthermore, the asymmetric and symmetric stretching bands of the amino group (-NH_2_) did not appear at 3600–3200 cm^-1^ . These results indicated a successful reaction, as expected.

**Figure 1:**
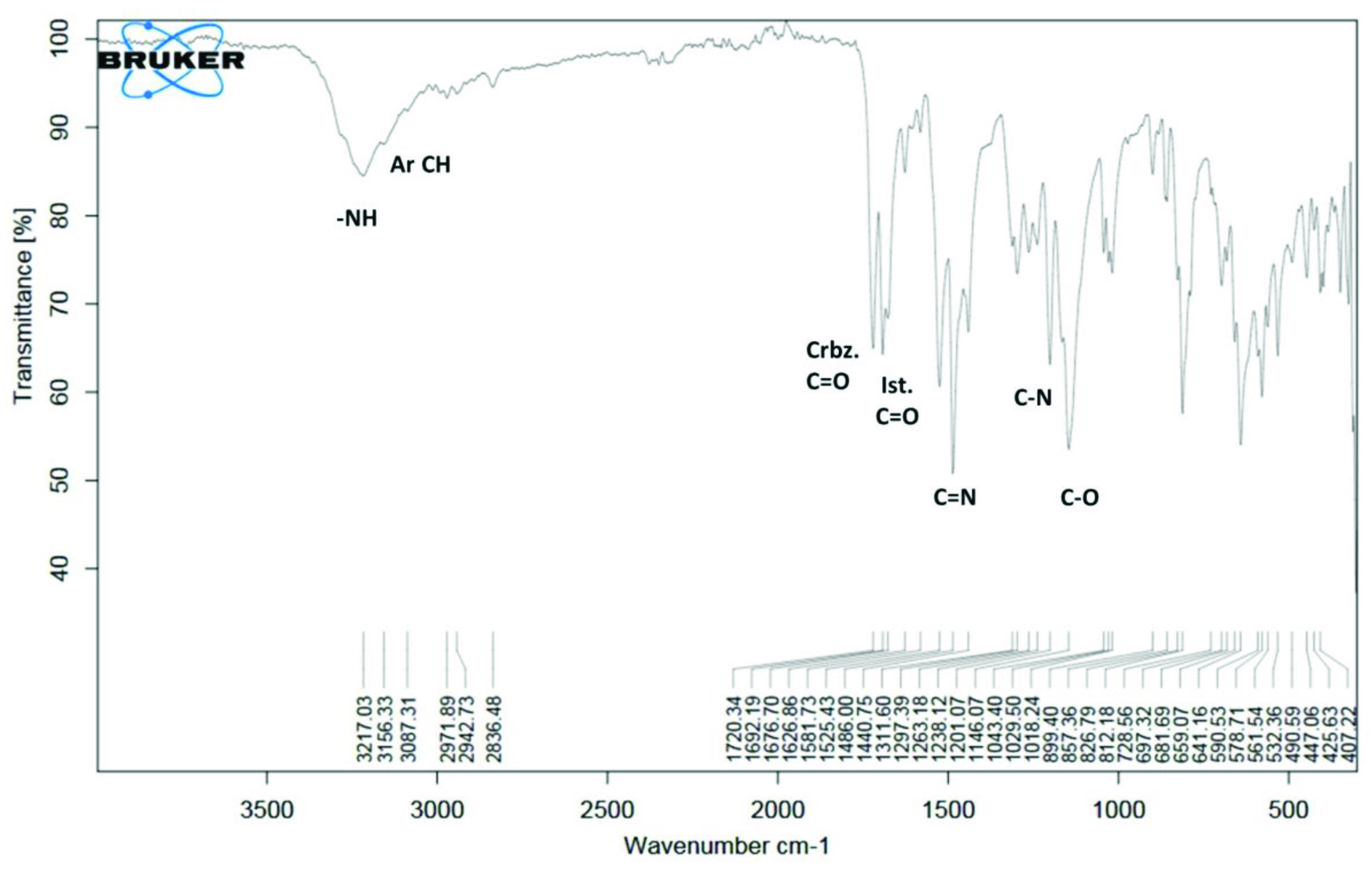
FT-IR spectrum of compound 6.

In compound 6, the -NH stretching vibration appeared at 3217 cm^-1^ . The C=O signals of the carbohydrazide region and the isatin ring were observed at 1720 and 1692 cm^-1^ , respectively. For compound 6, the -C=N stretching vibrations appeared at 1582 cm^-1^ , the -C-N stretching vibrations appeared at 1146 cm^-1^ , and the -C-O signals of the phenyl ring were observed at 1043 cm^-1^ , as shown in Figure 1. The IR peaks of the compounds are given in Table 3 (also see Supplementary information). The frequency values of all the synthesized compounds were in close agreement with those reported for similar compounds [6,32–34].

**Table 3:** Experimental FT-IR values of the compounds (cm^-1^) .

Comp.	-NH_2_	-NH	Ar CH	C=O	C=N	C=S	C-N	Spec. vib.
1	3392	3307	3199	1679	1595	-	1122	-
2	3324	3245	3106	-	1600	1554	1119	-
3	-	3142	3109	1729 1681	1591	-	1142	-
4	-	3213	3088	1736 1697	1593	-	1146	C-F: 879
5	-	3190	3104	1734 1683	1590	-	1136	C-Cl: 865
6	-	3217	3087	1720 1692	1582	-	1146	C-O: 1043
7	-	3132	2996	1693	1563	1503	1137	-
8	-	3128	2991	1689	1538	1499	1174	C-F: 872
9	-	3129	2992	1691	1539	1500	1165	C-Cl: 854
10	-	3156	3087	1692	1582	1486	1146	C-O:1043

### 3.3. ^1^H NMR spectral interpretations

**Figure 2:**
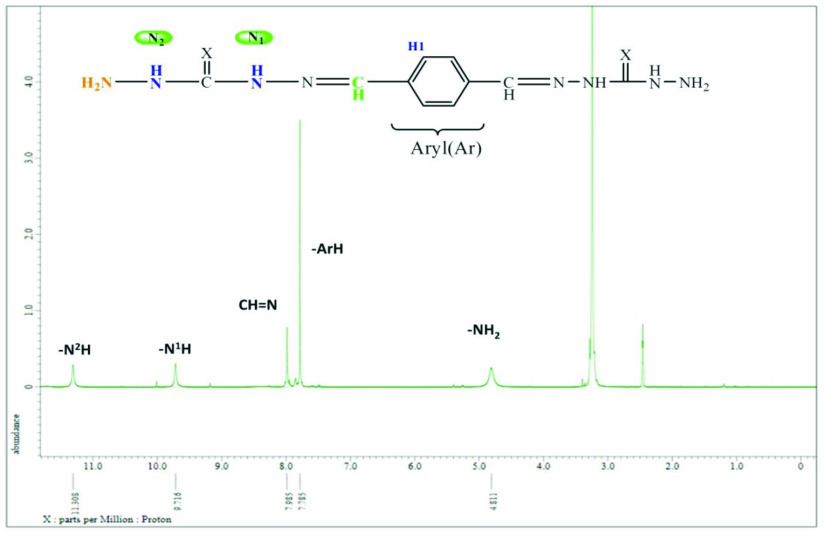
^1^H spectrum of compound 2.

The ^1^H NMR spectra of the synthesized compounds were detected in DMSO-*d_6_* as the solvent. For compound 2, the aromatic proton signal of the aryl ring (H1) was observed at 7.78 ppm (Figure 2). The signal of imine (–CH=N) was observed as a singlet at 7.98 ppm. Proton signals of carbohydrazide regions were observed from -N^1^H, -N^2^H, and -NH_2_ . These N -bonded proton peaks were observed as singlets at 9.72, 11.31, and 4.81 ppm, respectively.

**Figure 3:**
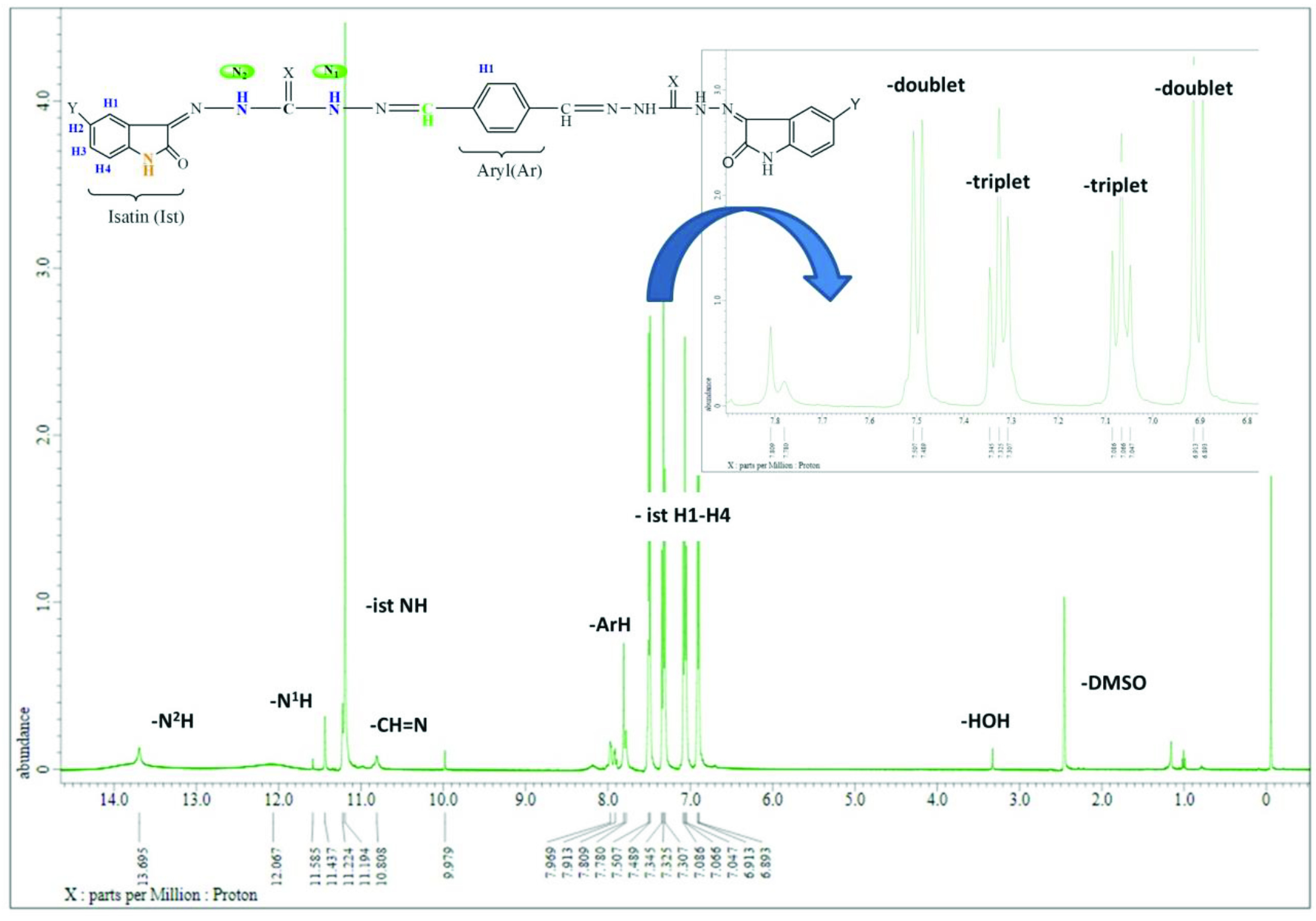
^1^H spectrum of compound 3.

In compound 3, the aromatic proton signal of the aryl ring (H1) was observed at 7.80 ppm (Figure 3). The signals of imine (-CH=N) were observed as singlets at 10.81 and 9.98 ppm. The -N^1^H and -N2 H proton signals of carbohydrazide regions were observed as singlets at 11.58, 11.44, 13.69, and 12.09 ppm, respectively. The -NH signals of isatin were observed as singlets at 11.22 and 11.19 ppm. The aromatic proton signals of the isatin ring (H1–H4) were observed between 7.51 and 6.89 ppm. The H1 proton coupled to the H2 proton and showed doublet peaks at 6.89 ppm. The H2 proton coupled to the H1 and H3 protons and showed triplet peaks at 7.08 ppm. The H3 proton coupled to the H2 and H4 protons and showed triplet peaks at 7.33 ppm. The H4 proton coupled to the H3 proton and showed doublet peaks at 7.50 ppm (Figure 3). Signals of DMSO-*d_6_* and water in DMSO (HOD, H2 O) were seen around 2.00, 2.50 (quintet), and 3.30 (variable, based on the solvent and its concentration) ppm, respectively [35–37]. These data are consistent with values reported earlier for similar compounds [6,32–34]. Proton chemical shift values of the synthesized compounds are given in Table 4.

**Table 4:** ^1^H NMR (δ , ppm, in DMSO-*d_6_*) values for synthesized compounds

Comp.	-NH_2_	-N2H-N	=N–N1H	CH=N	Ar H1	Ist NH	Ist H1	Ist H2	Ist H3	Ist H4	OCH3
1	4.00	10.64	10.25	7.83	7.67	-	-	-	-	-	-
2	4.81	11.31	9.72	7.98	7.78	-	-	-	-	-	-
3	-	13.69 12.09	11.58 11.44	10.81 9.98	7.80	11.22 11.19	6.89	7.08	7.33	7.50	-
4	-	13.75 13.36	11.63 11.48	9.98 8.70	8.13	11.23 10.80	6.89	-	7.23	7.85	-
5	-	13.68 11.50	11.37 11.29	8.73 7.97	7.79	10.81 9.98	6.91	-	7.35	7.45	-
6	-	13.85 11.94	10.98 10.80	8.18 8.17	7.71	9.98 9.96	6.74	-	6.84	6.97	3.70
7	-	14.57 12.65	12.00 11.69	8.66 8.19	7.91	11.33 11.15	6.93	7.07	7.35	7.55	-
8	-	14.82 14.51	12.70 11.29	8.73 8.52	8.12	10.66 9.95	6.87	-	7.21	7.86	-
9	-	14.87 14.50	12.83 12.73	10.02 9.99	8.17 7.89	11.51 11.42	7.48	-	6.93	7.38	-
10	-	14.91 14.51	12.82 12.55	8.62 8.36	7.84	11.19 11.08	6.75	-	6.85	7.00	3.72

### 3.4. ^13^C NMR spectral interpretations

**Figure 4:**
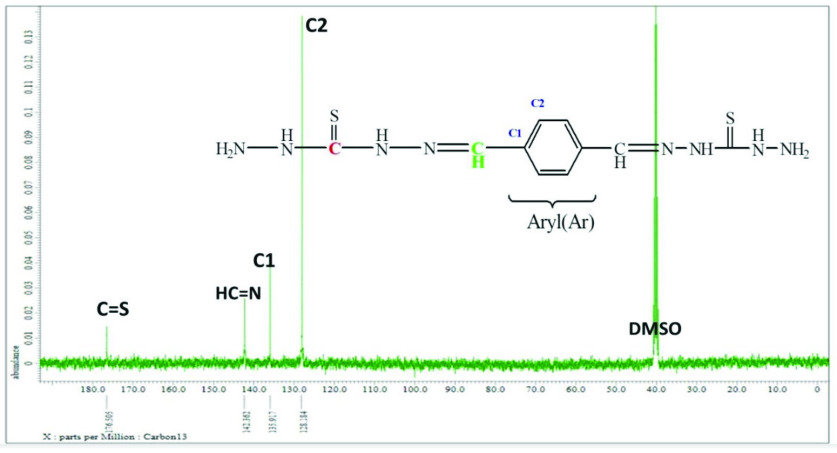
^13^C spectrum of compound 2

The ^13^C NMR spectra of all compounds were obtained in DMSO-*d_6_* . The ^13^C NMR spectrum of compound 2 showed 4 different resonances in good agreement with the proposed structure, as shown in Figure 4. In compound 2, the -C=S signal of the carbohydrazide region was detected at 176.5 ppm. The characteristic -CH=N (imine) peak was observed at 142.4 ppm. The aromatic carbon C1 and C2 signals were observed at 135.9 and 128.2 ppm.

**Figure 5:**
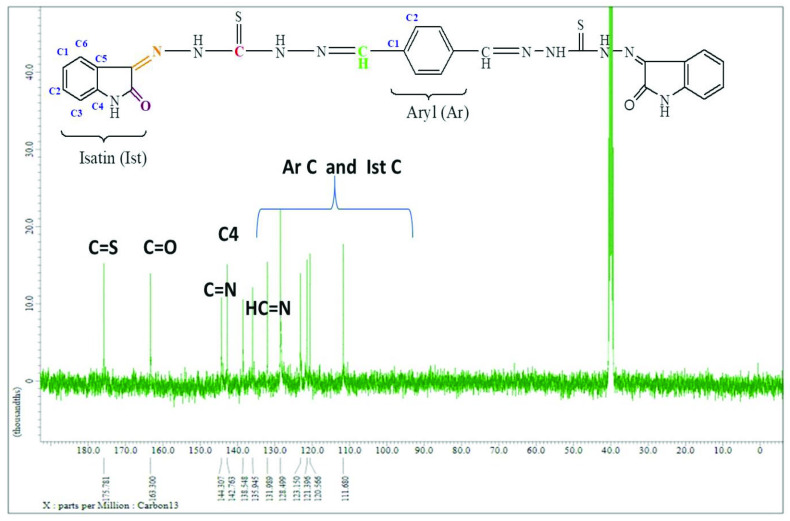
^13^C spectrum of compound 7.

For compound 7, the -C=S signal of the carbohydrazide region was detected at 175.8 ppm. The characteristic -CH=N (imine) peak was observed at 138.5 ppm. The aromatic carbon C1 and C2 signals were observed at 121.4 and 120.6 ppm, as shown in Figure 5. The characteristic -C=N and -C=O peaks of the isatin ring were observed at 144.3 and 163.3 ppm, respectively. The aromatic carbons (C1–C6) of the isatin ring were also observed at 135.9, 131.9, 111.7, 142.8, 128.5, and 123.2 ppm. Additionally, in compounds 4 and 8, the C atoms (for Ist C1–C6) were also split into doublets due to interacting with the atomic nucleus of F. These data are consistent with the values reported earlier for similar compounds [6,32,33]. The carbon chemical shift values of the synthesized compounds are given in Table 5 (also see Supplementary information).

**Table 5:** ^13^C NMR (δ , ppm, in DMSO-*d_6_*) values for synthesized compounds.

Comp.	C=O	C=S	CH=N	Ar C1	Ar C2	Ist C1	Ist C2	Ist C3	Ist C4	Ist C5	Ist C6	Ist C=N	Ist C=O
1	157.7	-	140.5	136.0	127.5	-	-	-	-	-	-	-	-	-
2	-	176.5	142.4	135.9	128.2	-	-	-	-	-	-	-	-	-
3	152.5	-	142.8	127.7	123.1	131.9	120.9	120.9	111.7	142.2	120.8	120.3	150.8	163.2
4	160.0	-	150.6	139.1	136.1	147.3 146.7	112.9 112.8	112.9 112.8	121.6 121.5	127.7 127.6	118.4 118.2	107.9 107.7	157.7	163.3
5	150.7	-	145.2	129.3	127.7	140.9	127.2	127.2	113.3	133.4	125.8	123.9	150.5	163.2
6	155.9	-	150.9	127.8	127.6	136.4	121.2	121.2	106.3	136.0	117.9	112.6	152.6	163.3
7	-	175.8	138.5	121.4	120.6	135.9	131.9	131.9	111.7	142.8	128.5	123.2	144.3	163.3
8	-	175.7	157.5	135.8	121.7	144.3 143.9	118.9 118.7	118.9 118.7	129.4 128.4	118.3 118.0	113.0 112.7	108.4 108.1	159.8	163.3
9	-	177.5	139.4	129.1	126.8	136.6	121.3	121.3	106.6	132.3	117.8	112.4	155.8	163.3
10	-	175.7	138.7	121.2	117.9	155.8	135.8	135.8	105.9	136.4	128.4	112.5	144.4	163.3

### 3.5. Evaluation of antioxidant activity

An antioxidant is defined as any substance that will delay or prevent the oxidation of a substrate, even when present at much lower concentrations than the oxidizable substrate [38,39]. In this study, IC_50_ values were calculated at the end of DPPH analysis for the synthesized compounds and for ascorbic acid, as shown in Table 6.

**Table 6:** IC_50_ values for the synthesized compounds.

Compounds	DPPH activity IC_50_ (μM)*
1	19.45 ±0.07
2	8.46 ±0.06
3	35.87 ±0.06
4	29.60 ±0.08
5	39.24 ±0.08
6	26.46 ±0.06
7	28.16 ±0.09
8	29.67 ±0.09
9	29.43 ±0.11
10	25.79 ±0.08
Ascorbic acid	12.33 ±0.05

*IC_50_ = Concentration (μM) exhibiting 50% inhibition of DPPH radical. Values are expressed as means (n = 3).

**Figure 6:**
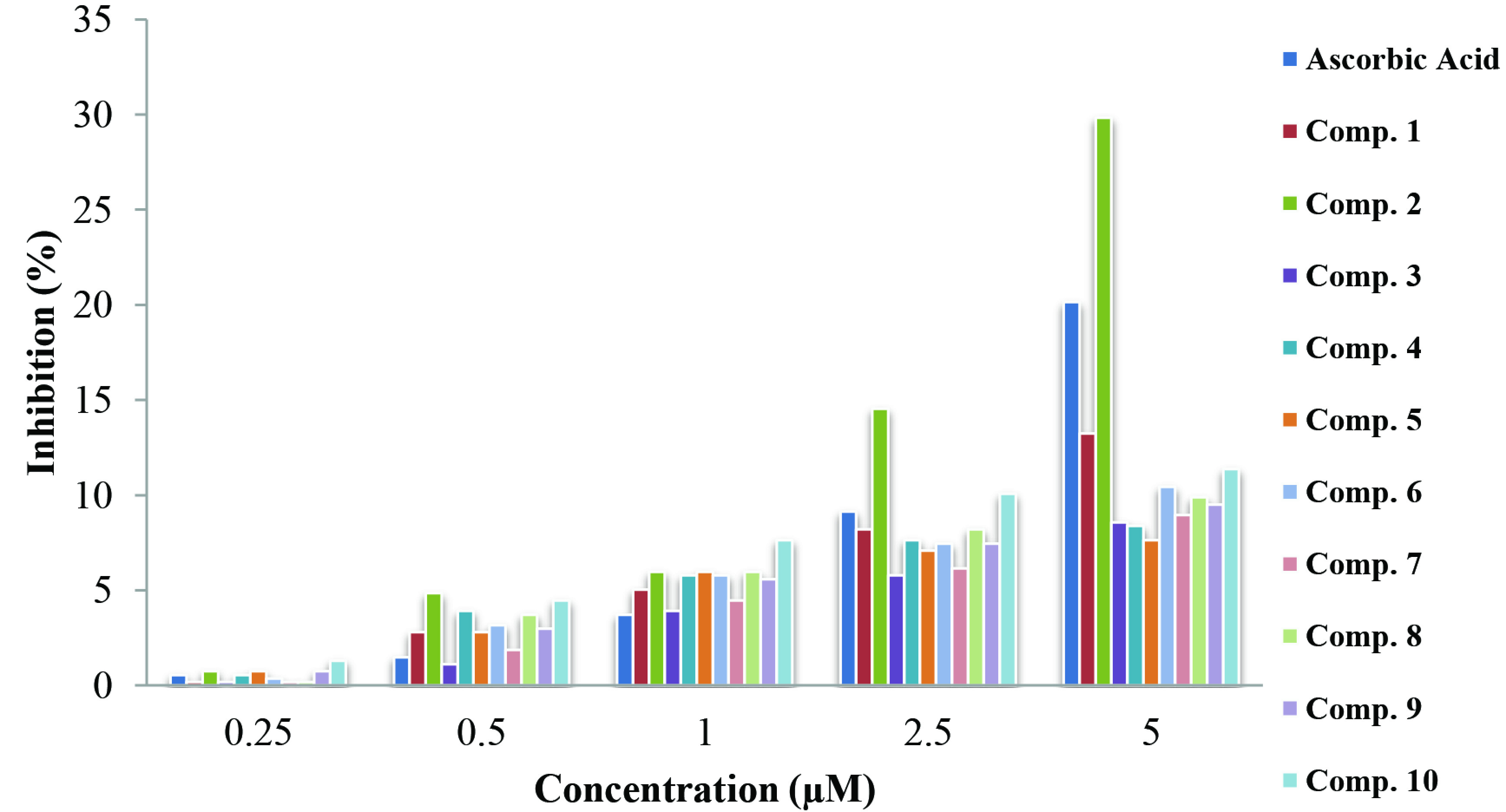
Variation of percent inhibition calculated by the DPPH method for ascorbic acid and the compounds at different concentrations.

The IC_50_ values of all synthetic test compounds were between 8.46 and 39.24 μM. Accordingly, compound 5 showed low activity, negligible in the DPPH assay. Compound 2 had the highest antioxidant activity. Except for compound 2, the compounds showed lower antioxidant activity than ascorbic acid (Figure 6).

Synthesized molecules 1 and 2 contain -NH_2_ groups at both ends in bilateral thio/carbohydrazone structures bound to the aldehyde group. Thus, these molecules have higher antioxidant activities in terms of IC_50_ values than other synthesized molecules. As in the review of Pakravan et al., N-H and -NH_2_ groups were responsible for the initiation of free radical scavenging activity [40]. In addition, it was observed that molecules having oxy groups showed lower antioxidant activity than molecules having thio groups [11]. Moreover, thiocarbazones have been utilized by many researchers due to their various biological activities. The sulfur atom also plays an important role in the biological activities of thiocarbazones. It was observed that synthesized compounds including sulfur atoms generally have higher antioxidant activities than other synthesized molecules.

In this study, it was observed that isatin structures containing different substituted groups bound to -NH_2_ structures partially decreased antioxidant activity. The presence of electron-withdrawing groups such as halogens on the aromatic ring has been observed to aid antioxidant activity [41]. The antioxidant activities of molecules containing H, F, Cl, and OCH3 groups bound to isatin were found in the order of -OCH3 >-F >-H >-Cl in the thiocarbohydrazone group and in the order of -OCH3 >-H >-Cl >-F in the carbohydrazone group, respectively. Previously, it was reported that methoxy groups in phenolic compounds increase antioxidant activity [30,42]. In this work, it was found that -OCH3 groups showed higher antioxidant activity compared to halogen group bound compounds because of the ability to provide electrons to the ring for both structures.

## 4. Conclusions

Some new bis-isatins and thio/carbohydrazones based on Schiff bases have been synthesized with excellent yields of 73%–98%. All the products were characterized by ^1^H NMR, ^13^C NMR, IR, and elemental analyses. The in vitro antioxidant properties of the synthesized compounds were measured by the DPPH free radical scavenging method. The best antioxidant activity was found for compound 2.

Supplementary Materials
