# Supplementary material for: Synthesis, spectroscopic studies, and antioxidant activities of novel thio/carbohydrazones and bis-isatin derivatives from terephthalaldehyde
Source: Turk J Chem. 2020 Feb 11;44(1):237–48. doi: 10.3906/kim-1910-13 (PMC7751813; doi:10.3906/kim-1910-13)
Supplement: Supplementary file 1 — Supplementary Materials [file turkjchem-44-237-sup001.pdf]

Supplementary information

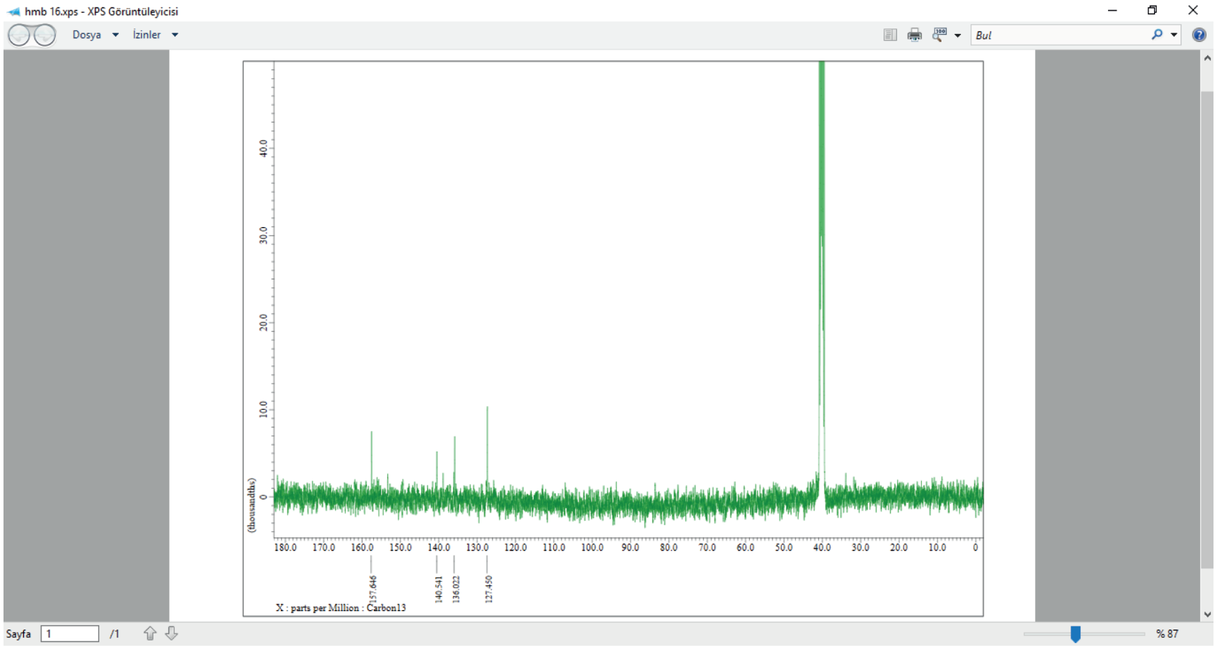

Figure S1. ^{13}C NMR spectrum of compound **1**.

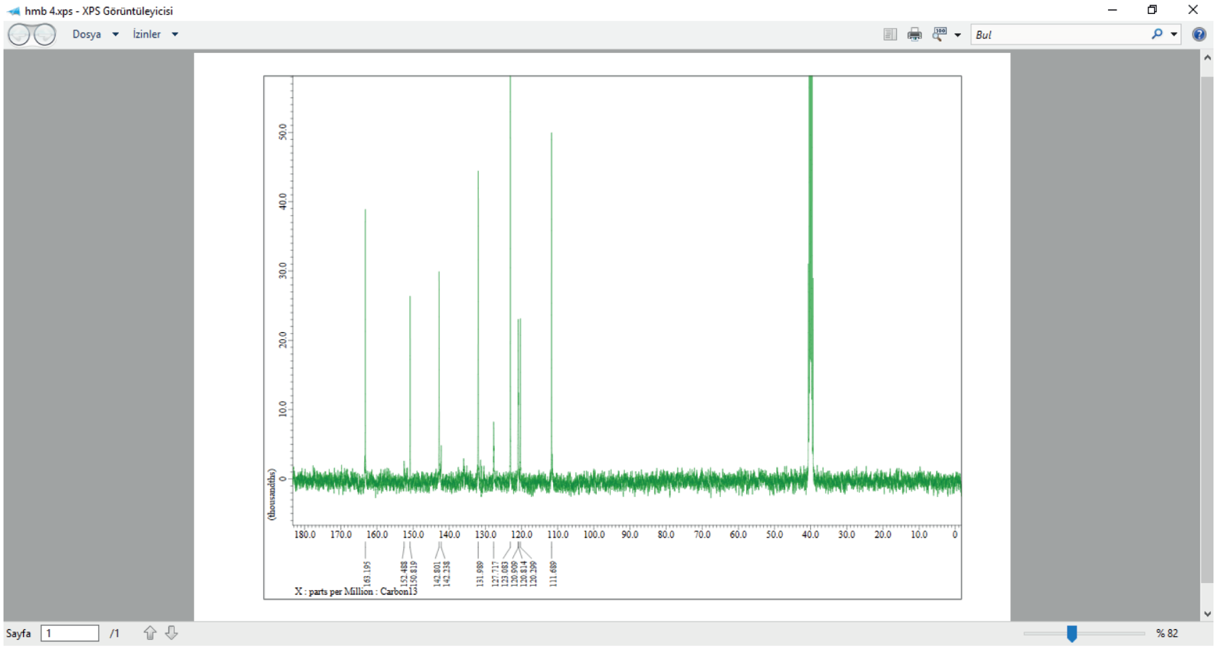

Figure S2. ^{13}C NMR spectrum of compound **3**.

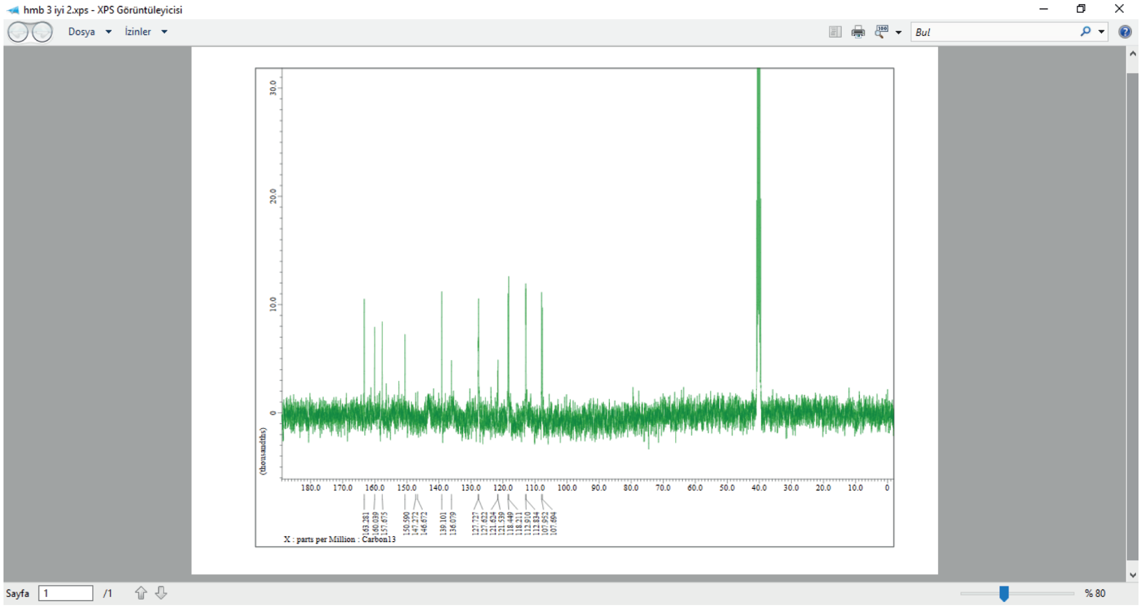

Figure S3. ^{13}C NMR spectrum of compound **4**.

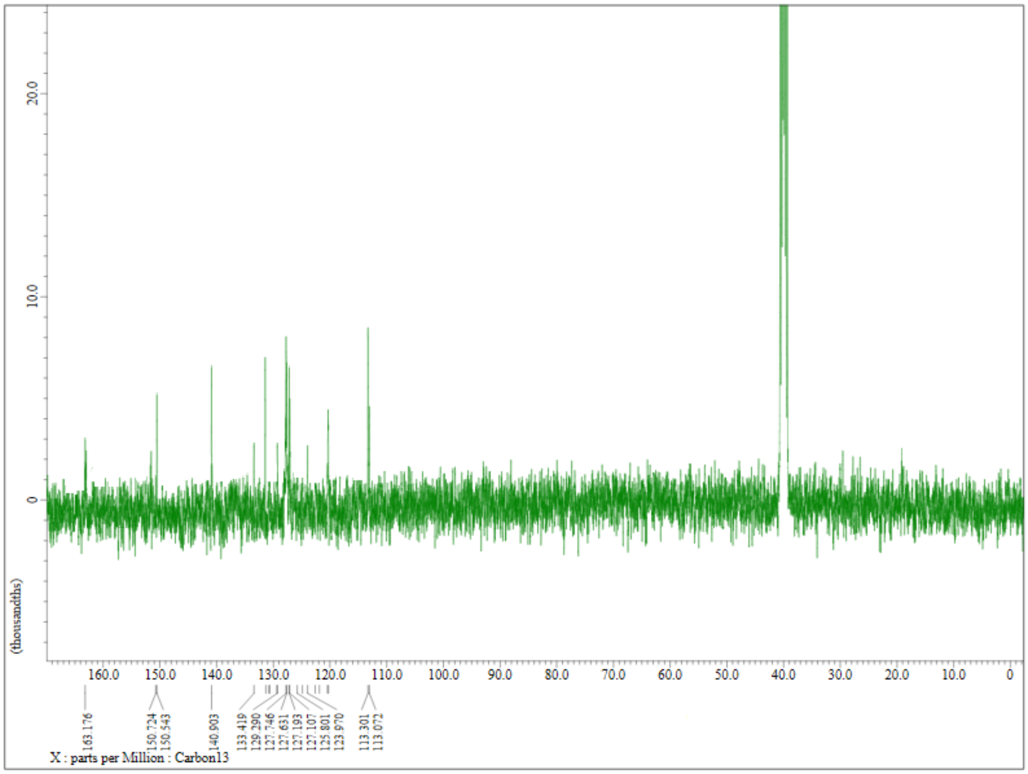

Figure S4. ^{13}C NMR spectrum of compound **5**.

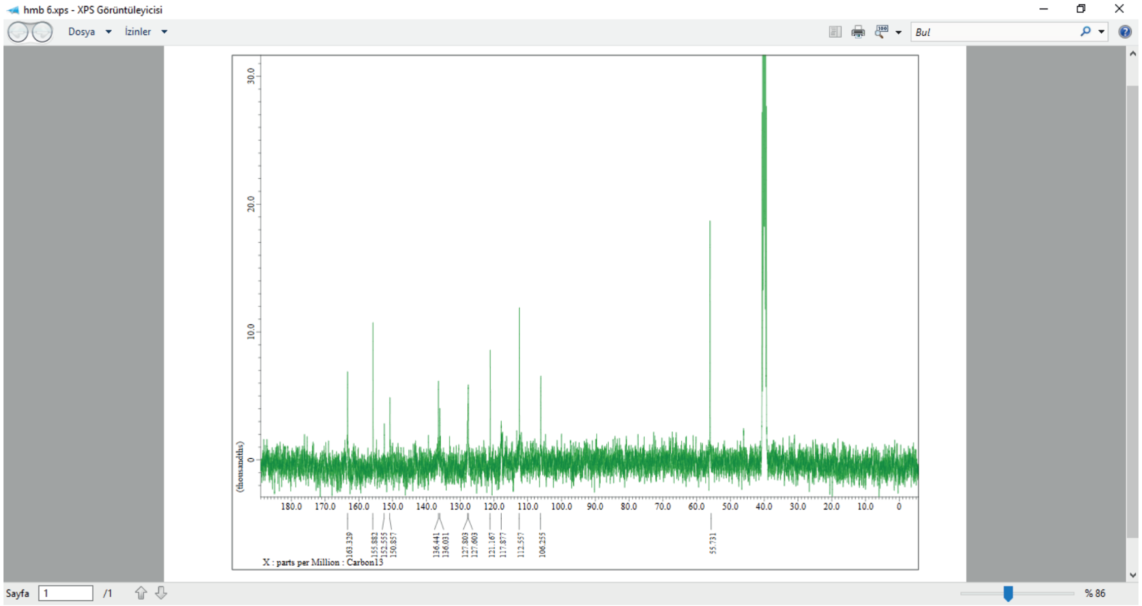

Figure S5. ^{13}C NMR spectrum of compound **6**.

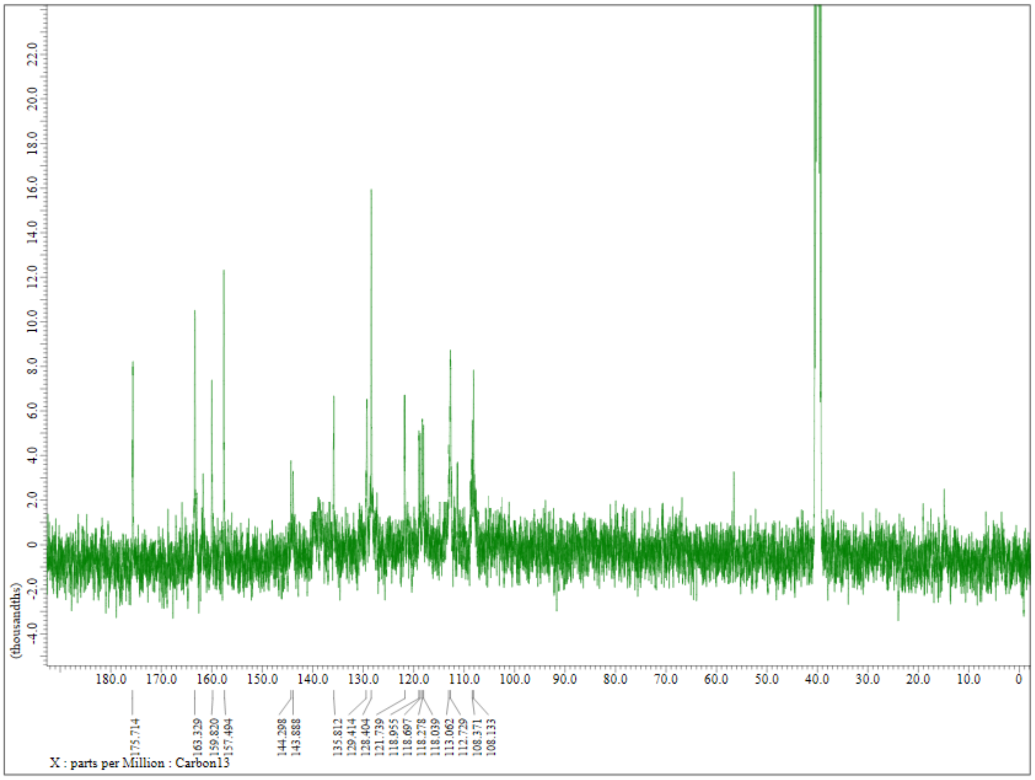

Figure S6. ^{13}C NMR spectrum of compound **8**.

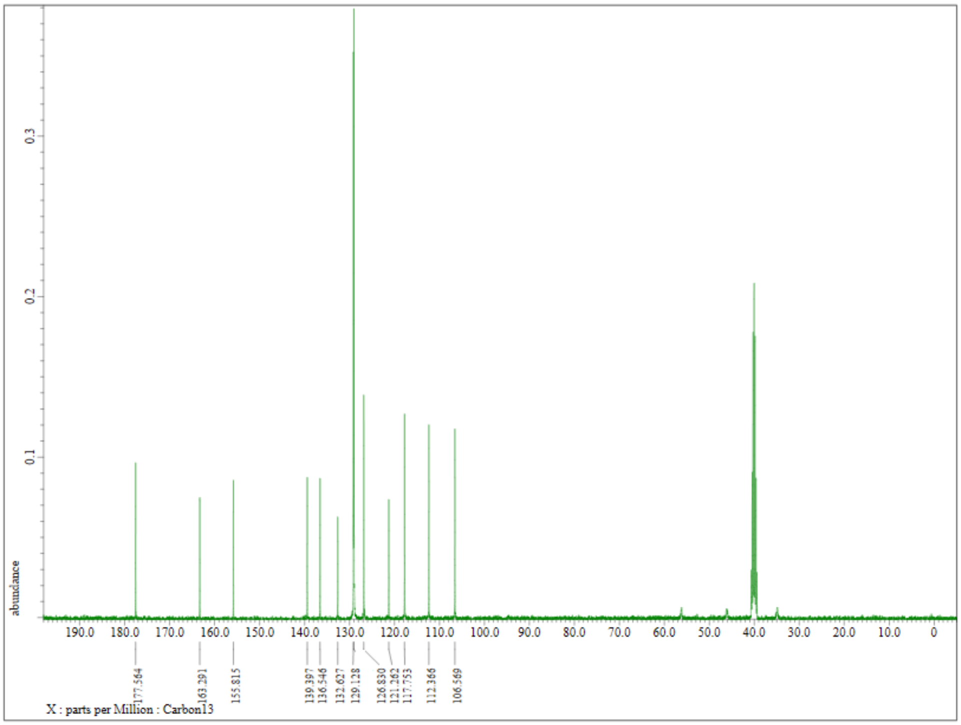

Figure S7. ^{13}C NMR spectrum of compound **9**.

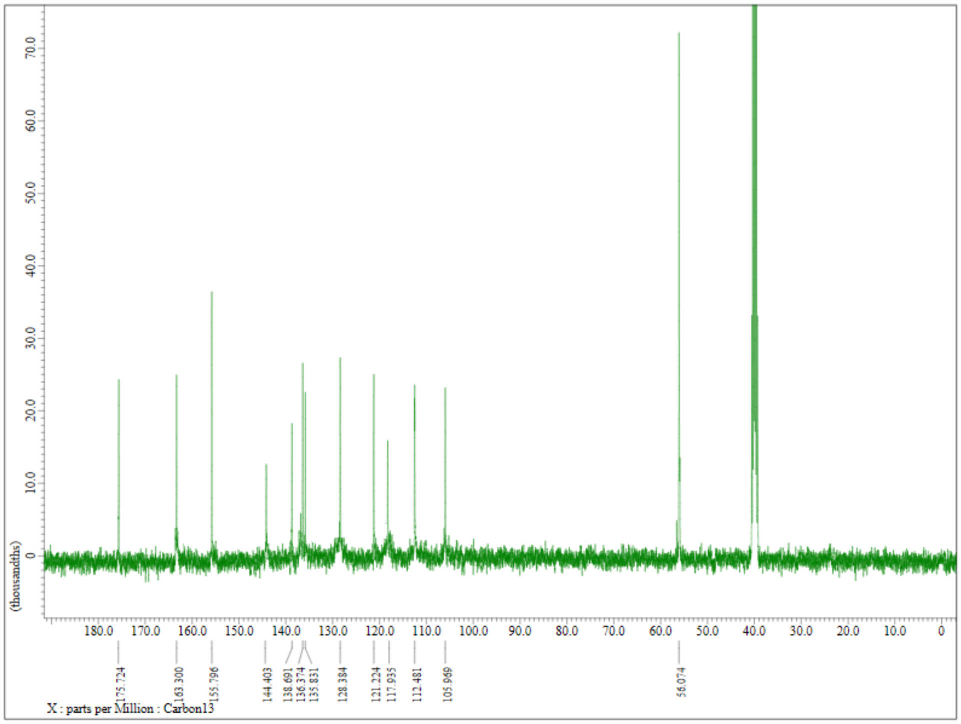

Figure S8. ^{13}C NMR spectrum of compound **10**.

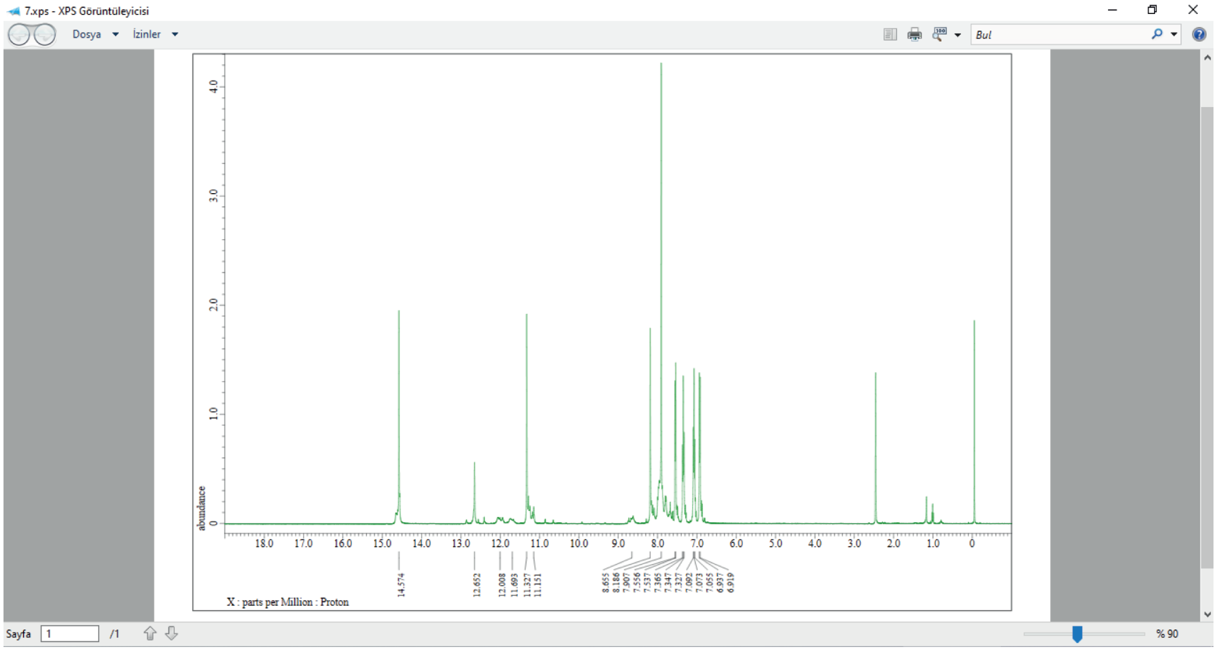

Figure S9. ^{1}H NMR spectrum of compound **7**.

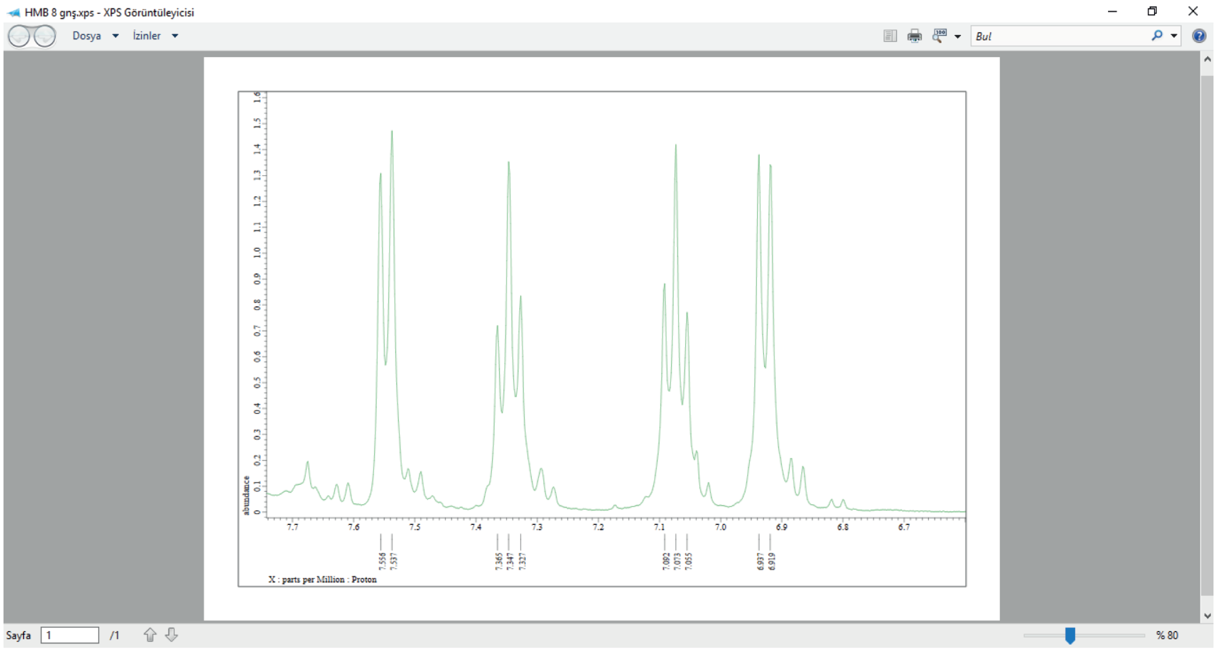

Figure S10. ^{1}H NMR extended spectrum of compound **7**.

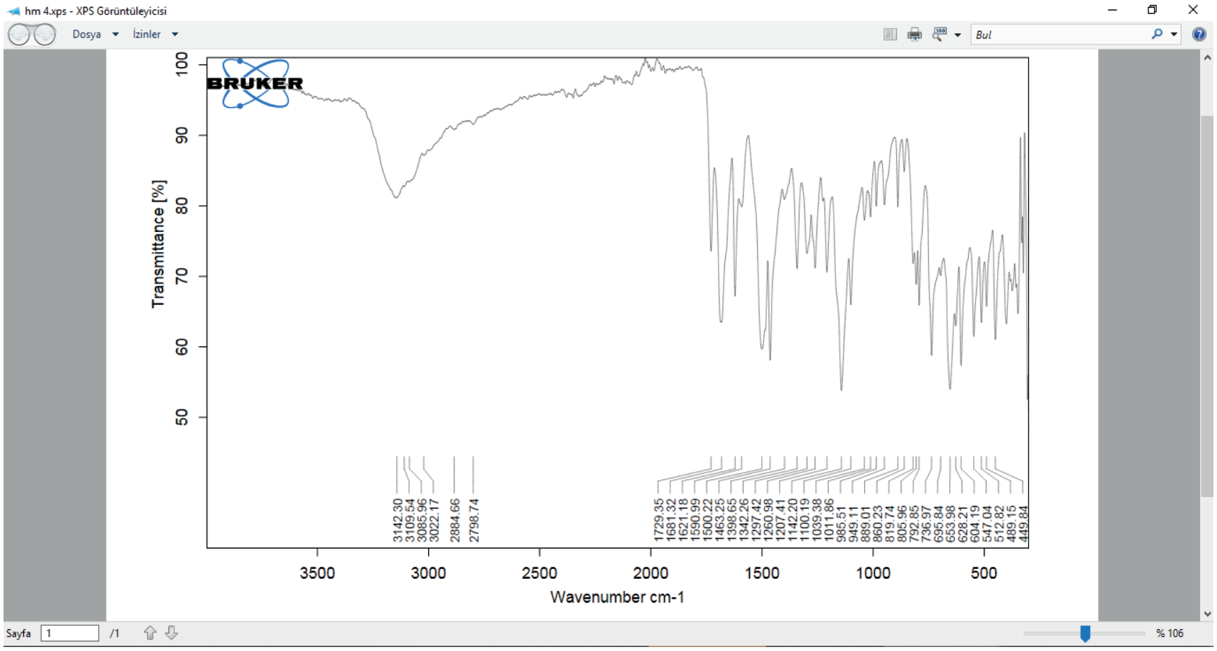

Figure S11. FT-IR spectrum of compound **3**.

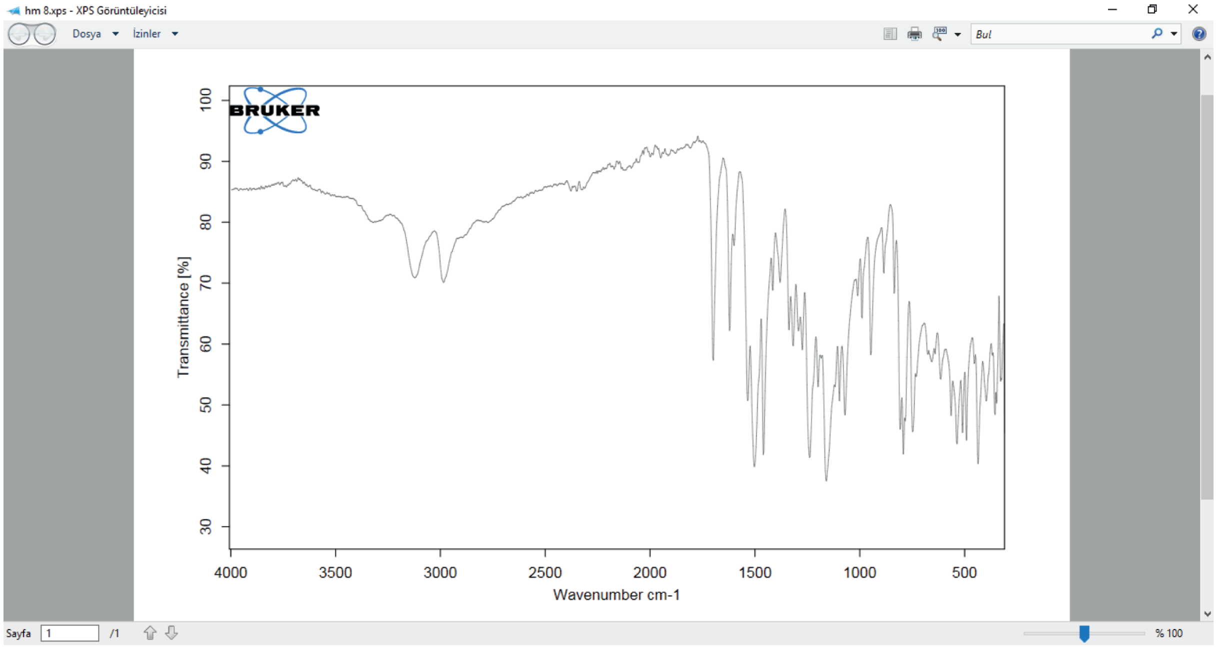

Figure S12. FT-IR spectrum of compound **7**.

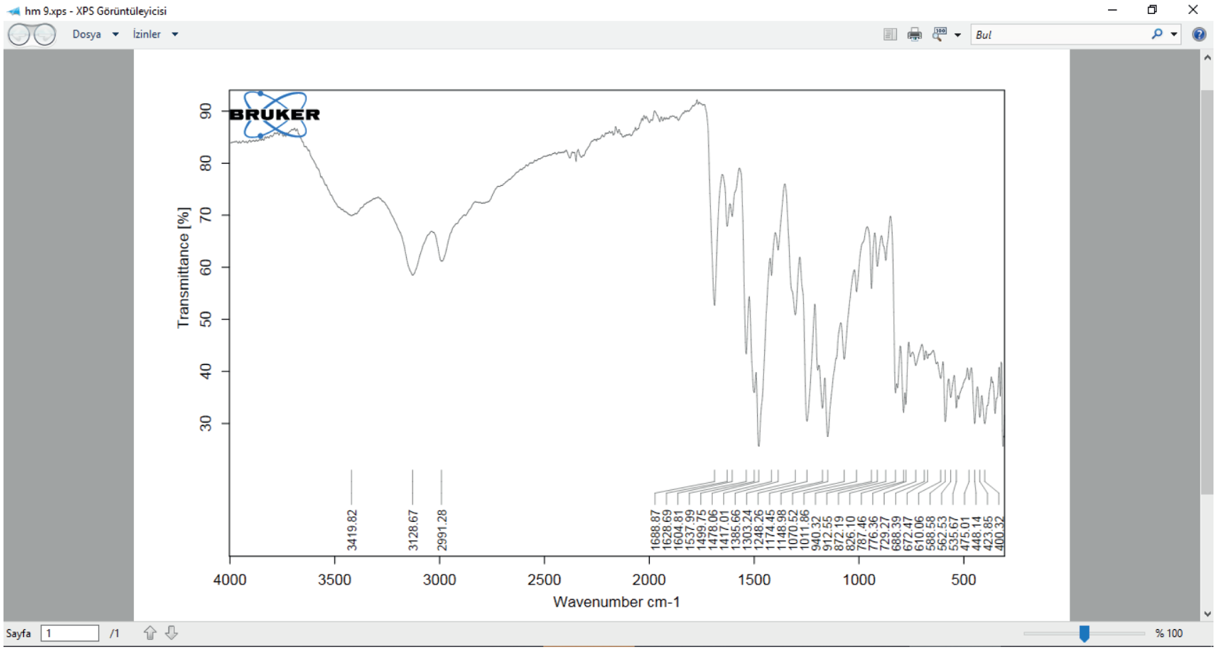

Figure S13. FT-IR spectrum of compound **8**.

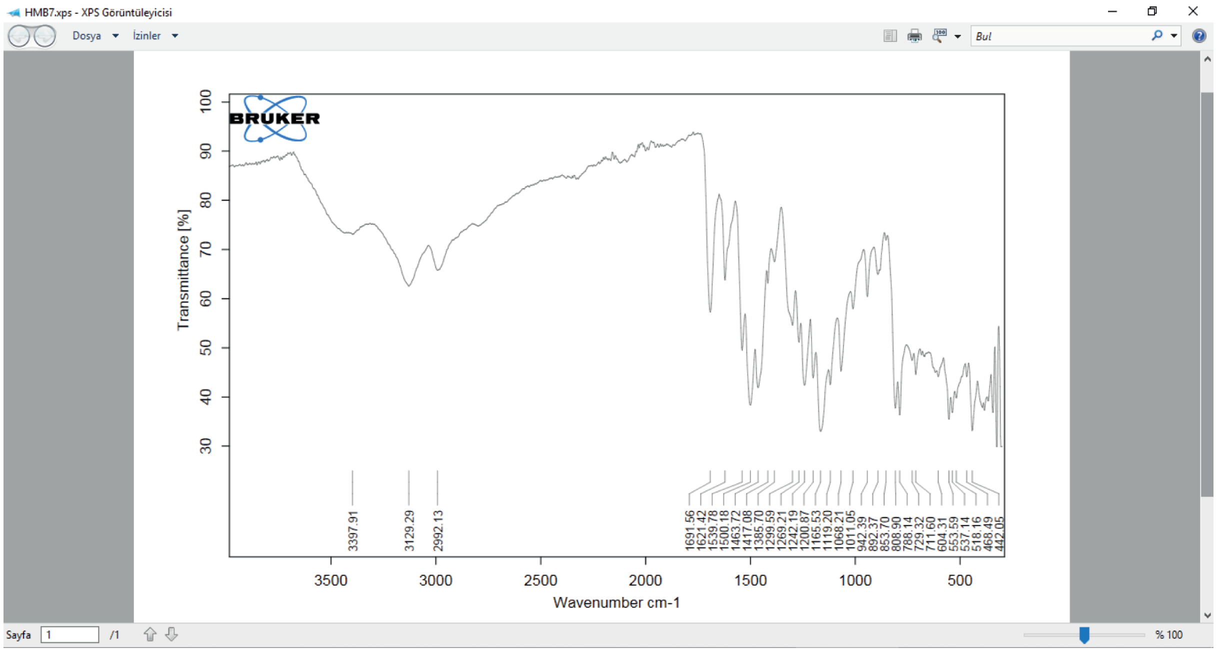

Figure S14. FT-IR spectrum of compound **9**.

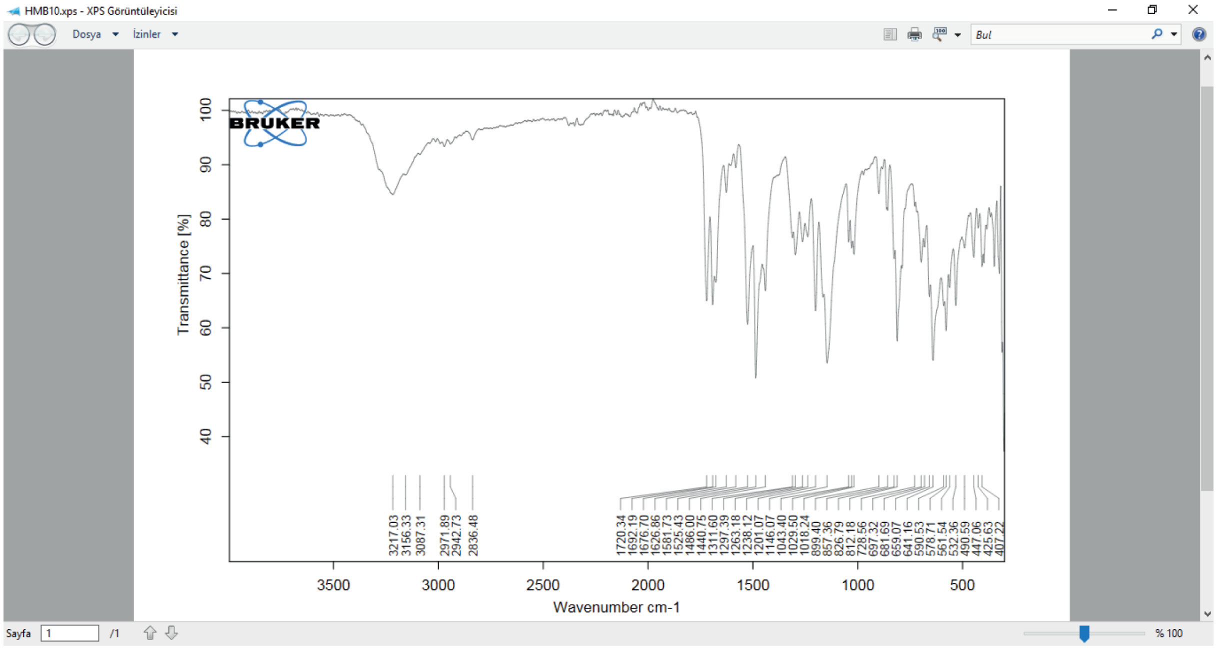

Figure S15. FT-IR spectrum of compound **10**.
